# The evolution of the national licensing system of health care professionals: a qualitative descriptive case study in Lao People’s Democratic Republic

**DOI:** 10.1186/s12960-017-0215-2

**Published:** 2017-08-07

**Authors:** Miwa Sonoda, Bounkong Syhavong, Chanphomma Vongsamphanh, Phisith Phoutsavath, Phengdy Inthapanith, Arie Rotem, Noriko Fujita

**Affiliations:** 1 JICA Project for Sustainable Development of Human Resources for Health to Improve MNCH Services, 2nd Floor, Sacombank Building, 044 Haengboun Rd, Ban Haisok, Chanthabouly District, Vientiane, Lao People’s Democratic Republic; 2 National Center for Global Health and Medicine, 1-21-1 Toyama Shinjyuku, Tokyo, 162-8655 Japan; 3 Ministry of Health, Ban thatkhao, Sisattanack District, Rue Simeuang, Vientiane, Lao People’s Democratic Republic; 4 University of New South Wales, 11/17 Sutherland Crs, Darling Point, Sydney, NSW 2027 Australia; 5 Japan Agency for Medical Research and Development, 1-7-1 Otemachi, Chiyoda, Tokyo, 100-0004 Japan

**Keywords:** Health policy, Health workforce, Licensing, Lao People’s Democratic Republic

## Abstract

**Background:**

The introduction of a systematic framework for the licensing of health care professions, which is a crucial step in ensuring the quality of human resources for health (HRH), is still evolving in Lao People’s Democraic Republic. The aim of this study was to review and document the evolution of Lao HRH policies and the development of its national licensing system.

**Case presentation:**

A qualitative descriptive case study methodology was applied to document and describe how Lao People’s Democratic Republic laid the foundation for the development of a licensing system.

The results demonstrate that Lao People’s Democratic Republic is currently in the process of transitioning the focus of its HRH policies from the quantity and deployment of services to remote areas to improvements in the quality of services. The key events in the process of developing the licensing system are as follows: (1) the systematic development of relevant policies and legislation, (2) the establishment of responsible organizations and the assignment of responsible leaders, (3) the acceleration of development efforts in response to the Association of Southeast Asian Nations Mutual Recognition Arrangement for standard qualifications, (4) the strengthening of educational systems for fostering competent health care professionals, (5) the introduction of a 3-year compulsory service component in rural areas for newly recruited government servants, and (6) the introduction of a requirement to obtain a professional health care certificate to work in a private hospital. The Lao Ministry of Health (MOH) has endorsed a specific strategy for licensing to realize this system.

**Conclusion:**

The need for licensing systems has increased in recent years due to regional economic integration and a shift in policy toward achieving universal health coverage. A national licensing system would be a significant milestone in health system development, helping to ensure the competency of health care professionals by means of a national examination, continuing professional development, and the revoking of licenses when appropriate.

## Background

The certification of health care professionals is a crucial step in the development of a modern health care workforce. Certification marks the successful completion of a process of assessment and verifies the competence and suitability of an individual to be recognized as a health care professional [1]. This recognition entitles the individual to provide health care within defined professional standards that legally mandate or prohibit certain activities. In most countries, it is illegal for health care practitioners to conduct medical practice without a valid license [2]. The formulation and implementation of standards and procedures for professional licensing require a legal and institutional framework that commonly involves designated health authorities, professional organizations, and other stakeholders such as educational institutions and the community at large [3]. Not only should the professionals’ competences and knowledge be regulated but the quality and safety of care needs to also be ensured.

Increasing attention is being paid to the need to ensure both the quality and safety of the public and private health care systems, thereby accentuating the need for a strong regulatory and institutional framework for the licensing of health care professionals. The World Health Organization (WHO) recognized human resources for health (hereafter referred to as HRH) as a key building block for better health, emphasizing the availability, accessibility, acceptability, and quality of the health care workforce as essential conditions in attaining universal health coverage (UHC) [4, 5].

The WHO’s “Global Strategy on Human Resources for Health 2030” also acknowledges the following as one of its objectives: “To optimize performance, quality and impact of the health workforce through evidence-informed policies on human resources for health” [6]. However, putting a policy and regulatory framework in place through a licensing system to ensure the quality of a health care workforce is often a neglected area in the development of HRH systems [7].

The Lao People’s Democratic Republic is a developing country in Southeast Asia with an estimated population of 6.2 million, 32% of whom live in urban areas. While Laos is a low-income country, its economy has been growing steadily in recent years [8]. The minimum threshold of 2.3 doctors, nurses, and midwives per 1000 population is considered necessary to deliver essential maternal and child health services; however, Lao People’s Democratic Republic still suffers from critical shortages in terms of the quantity and quality of HRH, having only 1.3 doctors, nurses, and midwives per 1000 population [9, 10]. This situation is also observed in neighboring countries, with thresholds of 1.3 in Vietnam, 1.3 in Myanmar, and 1.0 in Cambodia per 1000 population, countries categorized as being among the 49 countries with a critical shortage of health workers [10]. Therefore, priority has been placed on achieving a sufficient number of health workers [11]. However, Lao People’s Democratic Republic still lacks the mechanisms to ensure the quality of health care services provided by its health workforce. This is due to the absence of provisions and regulations that require health care professionals to be licensed based on competence certifications and requirements to regularly engage in continuing professional development as a condition for maintaining their license.

Providing quality and equitable health care services for all citizens has been a priority of the Lao government since the Law on Health Care was enacted in 2005 [12]. Although relevant legislation and policies have been formulated to enable the development of a licensing system over the last decade, determining whether such measures have been utilized effectively and what additional steps may be required to promote effective implementation remains difficult. No studies have examined the efforts made and constraints encountered in the development of these policies, and only a small number of officials have an accurate overall perspective of these efforts.

Therefore, the aim of this study is to examine the evolution of HRH policies in Lao People’s Democratic Republic with particular attention on the development of a regulatory framework, including a licensing system. To examine how Lao People’s Democratic Republic laid the foundation for the development of a licensing system, this review is designed to impart valuable lessons and useful insights into the development and implementation of a sound regulatory framework capable of ensuring the quality of the Lao health care workforce and its contribution to the health care system.

## Case description

A qualitative, descriptive case study methodology was applied to review the evolution of current policies and the development of a regulatory framework for a licensing system for health care professionals to improve its future implementation. This qualitative, descriptive case study design was officially approved by the Lao Ministry of Health. A document review was carried out of laws, regulations, policies, and relevant reports related to health care professionals in Lao People’s Democratic Republic. The study information was obtained from officially published documents including 209 Laotian laws, regulations, policies, and guidelines related to health that were issued between 1995 and 2016. Among these, 75 were categorized as “education,” 59 as “role and practice of health care professionals,” 2 as “qualifications,” and 73 as “others.” We found that 19 key documents were critical for the licensing system, and we ordered them chronologically (Table 1). Authors who had offered support in developing a licensing system strategy identified some key events in the evolution of the licensing system. Interviews were conducted with four key stakeholders who had also been involved in developing a licensing system strategy to collate and validate our observations from the document review and to ask what factors were important in the development of a licensing system. They consisted of senior officials from relevant departments in the Ministry of Health and vice deans from the faculty of medicine and dentistry at the University of Health Sciences. Interviewees agreed upon the conditions to remain anonymous and to have the confidentiality of their information rigorously maintained. All participants provided oral informed consent. Interviews were dictated without notation of the speaker’s identity. In addition, discussion notes and meeting memos that were obtained in a series of committee meetings to develop a licensing system strategy were utilized.

**Table 1:** Evolution and chronology of HRH and related issues in the development of a licensing and registration in Lao People’s Democratic Republic

Year	Main policies, strategies, and legislation of the licensing system for health care professionals	Description
2005, Dec	Law on Health Care (No. 09/NA/2005) [12]	◆The first law that determined the principles, regulations, and measures relating to the organization, activities, management, and control of health care activities.
		◆It defines 16 categories of health care professionals and states the associated requirements and responsibilities.
		◆The role of the Healthcare Professional Council was specified as the “secretariat for the Ministry of Health in administering the activities of health care professionals.”
2006, Dec	ASEAN MRA on Nursing Services [19]	Recognition, qualifications, and eligibility of foreign nurses were agreed upon by member states.
2007, Jan	Ministerial Agreement on the Healthcare Professional Council (No. 033/MOH/2007) [24]	◆The sub-regulation stipulates the details of Article 58 of the Law on Health Care; Rights and duties of the Healthcare Professional Council.
		◆ Article 2: The responsibility of the Medical Professionals Council
		The Healthcare Professional Council has to be the representative for the Minister:
		- To register certificates of those who wish to work as health care practitioners
		- To consider professional qualifications of health care professional applicants and bring to the Council committee meeting
		- To propose to the Minister of Health the official approval of health care professional certificates
		- To propose to the Minister of Health the approval of health care practices for foreign health care workers
		- To inspect the implementation of medical ethics and regulations to health care practitioners, particularly prohibited conduct as mentioned in Articles 36 to 43 of the Law on Healthcare
		- To supervise and inspect the continuing professional development of health care professionals
2007, Feb	Ministerial Agreement on Organize Health care Professional Council (No. 303/MOH/2007) [25]	According to the agreement on the organization of the Healthcare Professional Council, the Minister designates the membership of the Council, including the Chairperson, Vice Chairpersons, Commissioners, Secretary-General, and Vice Secretary.
2009, Feb	ASEAN MRA on Medical Practitioners [20]	Recognition, qualifications, and eligibility of foreign medical practitioners and so on were agreed upon by member states.
	ASEAN MRA on Dental Practitioners [21]	Recognition, qualifications, eligibility of foreign dental practitioners and so on were agreed upon by member states.
2011, Oct	The VIIth 5-Year Health Sector Development Plan (2011–2015) [39]	◆The 5-year plan aims to provide a clear road map for achieving MDGs and improving the health of the Lao people.
		◆Upgrading the staff’s level in terms of quality and quantity, in line with the Health Personnel Development Strategy, is planned under the Program of Human Resource Development.
2011, Nov	Health Personnel Development Strategy by 2020 (No. 831/MOH, Sep 28, 2010) [23]	◆The 10-year strategy aims to develop sufficient number of qualified health care personnel. It consists of the following 5 pillars: (1) health personnel capacity building, (2) utilization of health personnel, (3) health personnel management, (4) equity and equality of opportunity, and (5) health personnel incentives.
		◆Capacity development of the Healthcare Professional Council toward the realization of a licensing and registration system for health care professionals is targeted.
2012	Health Sector Reform Framework Laos 2013–2025 [16]	◆Health sector reform is a process concerning the approaches applied to improve the delivery of health services through existing systems in order to achieve the desired outcome of reaching MDG targets in Phase I (2013–2015) and will contribute to and shape the reform process to achieve UHC in Phases II (2013–2015) and III (2021–2025).
		◆Priority areas are as follows: (1) human resources for health; (2) health financing; (3) governance, management, and coordination; (4) health service delivery; and (5) hospital management and health information systems.
		◆A licensing system, continuing professional development, and capacity development of professional councils are 1 of the objectives.
		◆- Phase I (2013–2015): aims to achieve MDGs and develop a solid foundation for the following phases.
		- Phase II (2016–2020): aims to ensure that essential health services of reasonably good quality are accessible and can be utilized by a majority of the population.
		- Phase III (2021–2025): aims to achieve UHC.
2012, Jan	Ministerial Agreement on the Stationing of New Graduates in Rural Areas (No. 103/MOH/23, Jan, 2012) [31]	3 years of compulsory service in a rural area is recommended for civil servant candidates in order to be eligible for the licensing examination.
2013, Mar	Ministerial Agreement on the Organization and Activities of Department of Health Care (No. 695/MOH, 19/Mar/2013) [26]	The Office of Healthcare Professionals was established in the Department of Health Care of the MOH to support the activities of the Healthcare Professional Council.
2013, Jun	Ministerial Agreement on National Competency for Licensed Nurses in Lao People’s Democratic Republic (No. 1132/MOH, 6 Jun. 2013) [27]	The 9 necessary competency domains and indicators for licensed nurses are defined.
2014, Apr	Decree on Private Hospitals (No. 151/Gov, 28 Apr. 2014) [22]	Holding a health care professional certificate is conditional for health workers who work at a private hospital.
2014, Oct	Revised Curriculum for Higher Diploma Nursing Program, based on nursing competencies [30]	The competencies are integrated in each relevant subject of the higher diploma nursing program (3 years).
2014, Dec	Law on Health Care (amended) (No. 58/NA/2014) [32]	Working experience more than 3 years, passed examinations, and a nomination from the Healthcare Professional Council were newly defined conditions for health care professionals.
2015, Jan	Ministerial Agreement on Medical Doctor Competencies (No. 095/MOH, 14 Jan. 2015) [28]	The necessary competencies for medical doctors are defined and expected to be integrated into the educational curriculum.
2015, Jan	Ministerial Agreement on Dentist Competencies (No. 095/MOH, 14 Jan. 2015) [29]	The necessary competencies for dentists are defined and expected to be integrated into the educational curriculum.
2015, Jan	The VIIIth 5-Year Health Sector Development Plan (2016–2020) (MOH/2015) [33]	A project for developing the licensing system for health care professionals is currently planned, and the necessary budget is under request.
2015, Dec	Strategy on Healthcare Professional Licensing and Registration System in Lao People’s Democratic Republic 2016–2025 (No. 2098/MOH, 3 Dec. 2015) [23]	The 10-year strategy for developing the licensing system specifies its goals, objectives, targets, prioritized actions, and framework.

*MOH* Ministry of Health, *MRA* Mutual Recognition Arrangement; *UHC* universal health coverage

In the following section, we present the chronology of the development of a licensing system in Lao People’s Democratic Republic from 2005 to 2015 (Table 1), followed by a description of the key events identified in the evolution of the system.

### The chronology of a licensing system in Lao People’s Democratic Republic (from 2005 to 2015)

In 2005, Lao People’s Democratic Republic enacted the country’s first “Law on Healthcare” to strengthen health governance toward achieving its health care goals [12]. It regulated 16 categories of health care professionals with a statement of required responsibilities. The role of the Healthcare Professional Council was also specified in Article 58 as the “secretariat for the Ministry of Health in administering the activities of health care professionals.” For these reasons, when examining legal and official document archives, we considered 2005 as the starting point of the system development.

The development of a legal framework started with the core health law and was followed by sub-decrees and other relevant legislation. Over the past decade, key policies and strategies designed to improve human resources for health have been introduced, which have resulted in minor inconsistencies and overlap in legislation.

**Fig. 1:**
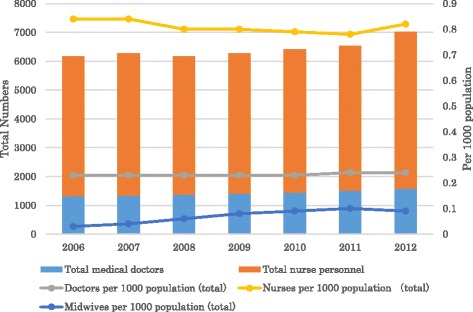
Total numbers of medical doctors and nurses in Laos, 2006–2012. Source: Lao People’s Democratic Republic Health System Review, 2014, Asia Pacific Observatory on Health System and Policies [13]

**Fig. 2:**
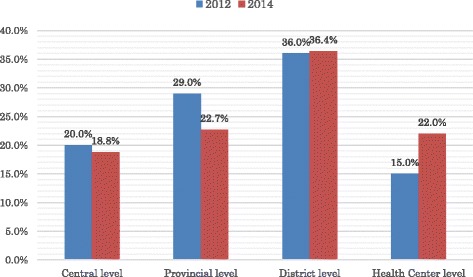
Distribution of health workers in Lao People’s Democratic Republic in 2012 and 2014. Source: Ministry of Health, Annual report on distribution of health personnel in 2012 and 2014 [14, 15]

During this period, HRH policy in Lao People’s Democratic Republic shifted from “strengthening the deployment of health workers in rural health care facilities” to “improving the quality of health care professionals.” Although Lao People’s Democratic Republic has taken active steps to strengthen HRH in terms of quality and quantity over the past few decades, the number of health workers working in the public sector remained virtually unchanged between 2006 and 2012. The total number of health workers stagnated at approximately 12 400, despite a 23% increase in population during the same period (Fig. 1) [13]. This was because of the limited absorption capacity of HRH in the public sector. To address critical health care workforce shortages, especially in rural areas, the Ministry of Health (MOH) has actively promoted the recruitment and retention of health workers. In addition, the Lao government recently promulgated a decree on placing new graduates in lower-tier health care facilities, especially in rural and remote areas. As a result, the percentage of health care workers at the health center level has increased, making basic health care services available to the general population (Fig. 2) [14, 15]. According to “The Health Sector Reform Framework Laos” [16], three phases of reform were applied to improve the delivery of health care services and achieve the desired outcomes. For reaching the health-related Millennium Development Goals (MDGs) in Phase I (2013–2015), actions to increase the size and deployment of the health workforce in rural and remote areas have been made a priority. In Phases II (2015–2020) and III (2020–2025), increased emphasis has been placed on the enhancement of HRH production in terms of both quantity and quality to ensure community access to health care services and improve the quality of service provision. By 2025, Lao People’s Democratic Republic will have a sufficient and sustainable workforce that is skilled, motivated, supported, and widely distributed to ensure access to health services in achieving universal health coverage (UHC).

In the chronology, some critical events that are related to the evolution of this licensing system are observed: (a) acceleration by the Association of Southeast Asian Nations (ASEAN) Mutual Recognition Arrangement (MRA) for Lao People’s Democratic Republic to have standard qualifications, (b) establishment of responsible organizations and assignment of leaders in the early stages, (c) emphasis on human resources for health in developing the health system in Lao People’s Democratic Republic, (d) strengthened educational systems to foster competent health care professionals, (e) 3 years of compulsory service in rural areas for newly recruited government servants, and (f) requirements for obtaining a professional certificate to work at a private health care facility. Following these developments, (g) Lao People’s Democratic Republic has finally formulated a comprehensive developmental strategy for its licensing system, which will be implemented soon.

### Key events in the evolution of a licensing system for health care professionals

The following milestones and key events describe the gradual evolution of the licensing system in Lao People’s Democratic Republic:

#### Acceleration by the ASEAN MRA for Lao People’s Democratic Republic to have standard qualifications

In general, the migration of health care professions in the region of Southeast Asia did not seem to correlate closely with the development of underlying legal schemes, educational systems, and quality standards [17]. Rather, especially in Lao People’s Democratic Republic, insufficient qualifications and language barriers had a major restraining effect on the migration of Laotian health care professionals abroad [18]. The MRA accelerated the production of health care professionals using a regional standard in Lao People’s Democratic Republic particularly by upgrading its educational system.

Even though there is a small but increasing number of international health care professionals in Lao People’s Democratic Republic, no clear policies had been formulated to regulate the qualifications of foreign health care professionals to practice in Laos until recently and no systems had been in place to monitor their practices [13]. However, the ASEAN MRA which encompassed nurses in 2006 and medical practitioners and dentists in 2009 [19–21] enforces member states’ reform of their regulatory frameworks by establishing procedures to regulate foreign health care professionals. Lao People’s Democratic Republic has initiated the necessary arrangements for foreign practitioners. For example, certification of foreign health care workers to work in private hospitals has recently been required by the council [22]. As integration into the ASEAN economic community (AEC) increases, the establishment of national standards according to the newly endorsed strategy is being accelerated, including plans to detail the necessary conditions for foreigners to obtain a Lao license [23].

#### Establishment of responsible organizations and assignment of leaders in the early stages

In 2007, 2 years after the adoption of the Law on Health Care [12], the MOH issued two sub-decrees. The first specified the role of the Healthcare Professional Council in terms of registration and the issuance of professional certificates [24], while the second stipulated that council representatives and additional official positions were to be allocated to designated leaders of the MOH [25]. Although the Healthcare Professional Council was established at an early stage of the evolution of the licensing system with a clear mandate, it was unable to fulfill its responsibilities due to its insufficient management capacity. In the absence of full-time technical staff, the designated senior leaders were unable to implement the detailed work necessary to establish the regulations and mechanisms for licensing and registration. In 2013, the “Office of Healthcare Professionals” was established in the Department of Health Care of the MOH to serve the secretariat and support the activities of the Healthcare Professional Council [26]. Even though the number of full-time dedicated staff remained limited, their contributions played a major role in the development of a detailed strategy for the development of the licensing system, which was finally enacted in 2015. However, the institutional framework is not functional yet.

#### Emphasis on human resources for health in developing the health system in Lao People’s Democratic Republic

The urgent need to establish a framework and mechanisms for monitoring and regulating the quality of health care professionals has been recognized by the MOH and was included as a priority in “The Health Personnel Development Strategy by 2020” which was approved by the prime minister in 2011 [11]. This strategy stipulated the urgent need to functionalize the Healthcare Professional Council and identified priorities to ensure its contribution. The strategy stressed the importance of the following:

- ○ Reviewing provisions to ensure that the Healthcare Professional Council and its boards had sufficient resources and expertise as well as an effective management structure to enable the successful undertaking of its responsibilities as stipulated in the Health Law with an emphasis on:
- ○ Approving the competency framework for certifying health care graduates;
- ○ Approving the methods and content of existing exams for health care graduates as a basis for licensing; and
- ○ Developing a framework and criteria for the licensing of health care professionals, including provisions for continuing periodical registration based on evidence of continuing professional development and practice.

#### Strengthened educational systems for fostering competent health care professionals

In addition to facilitating the cross-border movement of professionals, improving education and legislation related to health care professionals in Lao People’s Democratic Republic in the context of these new ASEAN initiatives has been emphasized. Professional competencies for medical doctors, dentists, and nurses were developed to provide specific criteria for their assessment by the MRA [27–29]. In addition, in line with international standards, the educational curriculum was revised based on these competencies, allocating more study units for professional subjects [30]. Moreover, to meet MRA requirements, in which only a professional nurse can be eligible for the MRA as opposed to a nurse with only technical qualifications, nursing education programs were extended from 2.5 to 3 years.

National examinations are planned to ensure the competency of graduates to practice in Lao People’s Democratic Republic. Although 10 health care educational institutions are currently under the jurisdiction of the MOH, an institutional quality assurance system has yet to be formally applied across all levels and categories of health care professionals including medical doctors, dentists, nurses, pharmacists, and midwives [11, 13]. It is anticipated that a national examination for medical doctors, dentists, and nurses will be implemented by 2020 and for others by 2025 [23]. As a sole exception, since the introduction of a national examination in 2010, midwives have been issued a certificate to allow them to practice [13]. The national examination includes an objective and structured clinical examination (OSCE) based on national midwifery education standards issued by the MOH and following the International Confederation of Midwives (ICM).

#### Three-year compulsory service in rural areas for newly recruited government servants in the health sector

Lao People’s Democratic Republic has suffered a critical shortage of HRH, and the inequitable distribution of HRH is particularly evident in remote and rural areas [9]. A shortage of HRH in a resource-limited country such as Lao People’s Democratic Republic is primarily caused by low production and employment capacity. The MOH cannot employ all nursing graduates (approximately 500–600 per year) or all graduates in other health professions due to the limited number of sanctioned posts in the public sector, which applies to almost all hospitals [13]. To address this critical issue in community health with a limited government budget, the MOH enacted the “Ministerial Agreement on the Stationing of New Graduates in Rural Areas (No. 103/MOH/23, Jan. 2012)” [31], which mandates 3 years of compulsory service in rural areas for all kinds of newly employed government servants in the health sector. Those who complete the 3-year compulsory service in rural areas are eligible to sit for the licensing examination. It is an incentive for placing staff in such areas. However, the development of a training program with a formal supervision scheme will be a challenge in the future.

The 3-year term of service is consistent with the requirement for licensure that mandates 3 years of clinical service before obtaining a full license [24, 32] (Fig. 1).

#### Requirements for obtaining a professional certificate to work at a private health care facility

Almost all hospitals belong to the public sector, but the number of private clinics is increasing. The Lao decree on private hospitals (no. 151/Gov, 28 April 2014) [23] was promulgated in 2014 and stipulates that a health care professional who intends to work at a private hospital must hold a certificate issued by the MOH. This is not the same as a professional license to prove competency of practice; in contrast, it provides official recognition for a health care practitioner who is not registered in the government servant registry to work in a private health care facility. Needing to control the quality of health care in the private sector, the MOH began issuing official certifications in 2014. However, the government of Lao People’s Democratic Republic intends for this stream to be integrated as a unique system of licensure for health care professionals to avoid the complexity of a potential dual system.

#### Ten-year strategy for the licensing and registration of health care professionals

In 2015, the MOH endorsed the “Strategy on Healthcare Professional Licensing and Registration System in Lao People’s Democratic Republic 2016-2025” [23]. This strategy ultimately defined the framework for the licensing and registration system and consisted of goals, objectives, targets, and prioritized actions over the next 10 years, with a focus on six pillars (Table 2).

**Table 2:** Framework of strategy for the licensing system for health care professionals

Goal	Health care professionals meet the professional standards required for licensing and registration as a health care professional in their respective discipline leading to improved health services and better health outcomes.					
Objective	Strengthening and implementing the regulatory framework and mechanism for licensing and registration of the health care professionals under the responsibility of the Healthcare Professional Council.					
Strategy	(1) Strengthen organizational capacity of the Healthcare Professional Council.	(2) Develop the legal and regulatory framework for the licensing and registration system for health care professionals.	(3) Implement national licensure examination for health care professionals.	(4) Formulate standard and procedures for issuing professional license and registering health care professionals.	(5) Develop a system for implementing continuing professional development (CPD) that meets the requirements for renewal of registration of health care professionals.	(6) Develop the information management system for licensing and registration of health care professionals and strengthen public communication.
Priority actions	1-1. Appoint the President, Vice President, and members of the Healthcare Professional Council and its professional Boards. 1-2. Revise the regulation of Healthcare Professional Council No:033-MOH Date 24 January 2007 (including its TOR, authority, and accountability). 1-3. Identify and provide required technical and training support for operating licensing and registration system for health care professional 1-4. Develop financial management system for operating the licensing system and registration for health care professional and identify funding sources and partnerships to ensure sufficient resources as may be required.	2-1. Establish committees and working groups to review, revise, and develop the legal and regulatory framework for the licensing and registration system in line with relevant legislations. 2-2. Make plan to implement the approved legal and regulatory framework for licensing and registration system for health care professionals 2-3. Disseminate and advocate the enacted legal and regulatory framework to promote the implementation of the licensing and registration system for health care professionals nationwide.	3-1. Set up National Examination Committees for all categories of health care professionals. 3-2. To formulate outline of the National Examination to certify competency for initial license, including the scope of examination, subjects and topics to be included, frequency of examinations, types of test (MCQ, OSCE, etc.), method of test (paper, computer-based, etc.), and the criteria passing score or rate. 3-3. Develop table of specification (blueprint) for each examination and design and pilot test with samples of target groups. 3-4. Manage the implementation of the national licensing examinations to certify competency for initial license in cooperation with the educational institutions with particular attention to the timing of the examination, the required logistic and supervision support, and the announcement of the test results.	4-1. Determine the process and standard for issuing professional licenses and registering to new graduates who graduated in Laos and foreign country, practicing health care professionals, and foreigner who is willing to work in Laos PDR. 4-2. Develop the process and standard for issuing professional licenses and registering guidelines and provide training for officers in charge of the administration of issuing license and registration of health care professionals. 4-3. Trial the procedures for issuing professional license and make necessary adjustments to ensure efficient and transparent application. 4-4. Manage the licensing and registration system according to the approved procedures and operating guidelines.	5-1. Determine the CPD requirements for renewal of registration including the type of learning activities and the number of credit points required within 5 years of registration. 5-2. Identify institutions and other groups which are providing CPD and explore what they could offer currently and in the future and the support they may require to optimize their contribution. 5-3. Develop criteria and standard for approval of CPD activities for renewal of registration. 5-4. Estimate the cost of implementation of CPD activities and define financial sources. 5-5. Make contract with approved institutions and health facilities for delivery of CPD activities and determine procedure for monitoring and evaluation. 5-6. Manage the renewal of registration according to the approved procedures and operating guidelines.	6-1. Develop and manage the national registry (database) for licensed health care professional. 6-2. Explore ways of integrating and/or linking the national registry (database) for licensed health care professional with existing health personnel and training databases maintained by the Department of Health Personnel and the Department of Training and Research to avoid unnecessary duplication and promote accurate and efficient information system. 6-3. Compile and disseminate annual reports, including statistics concerning the licensing and registration of health care professionals. 6-4. Disseminate and advocate information concerning licensing and registration through multimedia sources. 6-5. Monitor and evaluate the level of awareness and understanding concerning the licensing and registration system and take steps to address identified gaps to ensure better understanding.
Target by 2020	Organizational structure of the Healthcare Professional Council is clearly defined including TOR of all divisions and professional boards. Professional Boards for medical doctor, dentist, and nurse are established under the authority of the Healthcare Professional Council. Financial management system for operating the licensing system is established. Office of the Healthcare Professional Council is equipped with necessary equipment and facilities. Orientation and training is provided to the leadership and members of the Healthcare Professional Council.	Approved legal and regulatory framework for implementing the licensing and registration system for medical doctor, dentist, and nurse is implemented. Dissemination and advocacy workshops and other activities to promote the legal and regulatory framework are implemented in the central and provincial levels.	Document describing the procedures and mechanism is available and administration of licensing exams are in place. National licensing examination to certify competency for initial license is implemented for medical doctor, dentist, and nurse.	Completed issue of health care professional license for more than 80% of medical doctor, dentist, and nurse who work as clinical practitioners in private health care facilities. Completed issuing health care professional license for more than 60% of medical doctor, dentist, and nurse who work as a clinical practitioner in the governmental health care facilities. Completed issuing the health care professional license for more than 90% of foreign medical doctor, dentist, and nurse who work as a clinical practitioner in the private and public health care facilities (Practitioners with valid license of their country of origin). Completed 100% for issuing the temporary license for relevant foreign health care practitioners in private and public health facilities.	CPD trainings for medical doctor, dentist, and nurse are developed and approved by the Healthcare Professional Council. CPD trainings for medical doctor, dentist, and nurse are implemented under the guidance of the Healthcare Professional Council.	The national registry (database) for licensed health care professional is developed and operational multi-media communication tools for licensing and registration system are developed and applied. (website, poster, handbook, etc.).
Target by 2025	All other professional boards are established by the Healthcare Professional Council.	Approved legal and regulatory framework for implementing the licensing and registration system for all health care professionals are implemented. Dissemination and advocacy workshops and other activities to promote the legal and regulatory framework are implemented in the central and provincial levels.	Revised document describing the procedures and mechanism is available and administration of licensing exams is in place for all categories of health care professionals. National licensing examination is implemented for all categories of health care professionals.	Completed issuing the health care professional license for 90% of health care professionals who work as a clinical practitioner in the private health care facility. Completed issuing the health care professional license for 80% of health care professionals who work as a clinical practitioner in the governmental health care facilities.	CPD activities for all categories of health professionals are developed and approved by the Healthcare Professional Council CPD activities for all categories of health care professionals are implemented under the guidance of the Healthcare Professional Council. Registration renewal is started for health care professionals who meet the criteria.	An annual statistics report on health care professionals is published and disseminated

**Fig. 3:**
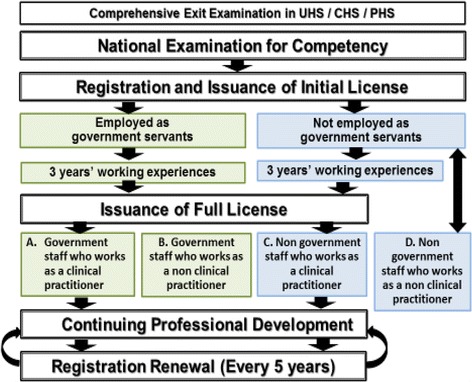
Framework of the licensing and registration system. Source: Strategy on Healthcare Professional Licensing and Registration in Lao People’s Democratic Republic 2016–2025 [23]

The pathway toward licensing and registration will commence with an exit exam administered by the training institution, leading to the awarding of an academic degree. Graduates who are awarded the degree are entitled to sit for a national competency examination that is administered under the supervision of the Healthcare Professional Council. To obtain a full license, candidates must complete 3 years of supervised practice in either public or private settings. To ensure that professional practice knowledge and skills are current, registered health care professionals are required to renew their registration every 5 years based on an assessment of their participation in continuing professional development (CPD) activities approved by the Healthcare Professional Council (Fig. 3).

In addition, the recently endorsed health sector development plan (the 8th, 2016–2020) [33] includes a national project for establishing a licensing and registration system. Based on these strategies, progress is now underway by the MOH in collaboration with the WHO to establish the Health Professionals Council to support this new system.

## Discussion

This qualitative, descriptive case study examined the evolutionary process of HRH policies in Lao People’s Democratic Republic by depicting the development of a regulatory framework for a licensing system. Introducing such a framework will be a significant milestone in the development of a Lao health care system and one that will have significant implications for the future. The ultimate goal of ensuring the quality of HRH is to provide safe and effective health care services and protect the population against malpractice. For example, licensure led to four major achievements in the US health care system: the elimination of quackery from unskilled interventions or delays in qualified care at the individual level, the reduction of infection by improved treatment, the advancement of science through improved knowledge and skills of medical practitioners, and cost savings from wasted payment to unskilled practitioners [34]. In Lao People’s Democratic Republic, the competency of health care professionals will be guaranteed by national examinations and continuing professional development. Moreover, by introducing a system in which licenses can be revoked because of malpractice, the ethical aspects of HRH and the importance of professional attitudes will be highlighted [23].

The need to develop a licensing system has accelerated in recent years not only in Lao People’s Democratic Republic but also in neighboring countries where no systems are in place. One of the primary factors in this development is the ASEAN MRA mandate for three categories of health care professionals, which was initiated at the end of 2015. Another factor is the increasing demand for control over private health care services, which have less administrative oversight [35] due to the international free flow of health services led by the ASEAN economic community.

Another driving force is the HRH policy shift from “strengthening the deployment of health workers in rural health care facilities” to “improving the quality of health care professionals.” Parallel with the increase of HRH deployment to rural health care facilities to achieve MDGs, licensing is essential to ensure the quality of HRH. The mere availability of health care workers is not sufficient; health care workers who are accessible and equipped with required competencies can deliver quality care that meets the expectations of the community and contributes to progress toward achieving UHC [6]. In the case of Japan, prior to introducing UHC, the quantitative aspects of health care workers were strengthened; furthermore, quality was improved by upgrading educational courses and licensing examinations [36]. This process is expected to be applied to other ASEAN countries that are still developing their licensing systems.

The development of effective policies and plans requires strong technical capacity to compile, analyze, and utilize HRH data, as well as the ability to draw upon best practices from abroad [37]. It is notable that there seem to be relatively few overlaps in the collected legal instruments and policies. In Lao People’s Democratic Republic, through strong top-down management as a sole regulator, both the long-term perspective and commitment of the MOH have played a substantial role in the systematic development and implementation of policies for health care professionals, as effective governance by the Healthcare Professional Council remains absent. As seen in other countries, the existence of multiple regulatory bodies, including the MOH and National Council, makes the system complex and only partially fulfills the tasks required for regulating health care professionals [38]. Although the responsibility to regulate and authorize health care professionals is usually attributed to the National Council in many countries, other authorities such as the Ministry of Health or other professional associations can take this role, or in some countries, the MOH staff concurrently belong to the Council [36]. To strengthen the Lao Council’s institutional capacity in the future, clear demarcations and effective collaboration between multiple stakeholders will be needed.

Even though Lao People’s Democratic Republic has started to develop a licensing system, some issues still need to be discussed carefully to reach consensus between different stakeholders. For example, special arrangements might be made for the national examinations of ethnic minorities. The latest census identified 47 distinct ethnic groups that constitute 48.5% of the total population, predominately in the highlands. This ethnic diversity still presents major challenges to health care delivery and education due to cultural and linguistic barriers [13]. Additionally, the licensing system for those who have graduated from foreign medical schools is under discussion as to whether to apply equivalent conditions to those who have graduated from medical schools in Lao People’s Democratic Republic. Additionally, the establishment of different license levels in the same professional categories, such as “Registered Nurse” and “Licensed Practical Nurse,” might be discussed and regulated in the future. One disadvantage regarding licensure is that it creates stringent barriers to becoming a health care professional. To supply sufficient numbers of health care professionals who serve the community, future licensure should conform to existing policies to address the actual demands in Lao People’s Democratic Republic.

When moving forward in countries such as Lao People’s Democratic Republic, it is necessary to transform health policy into practice by following strategies and action plans, including governmental budgets for strengthening the capacity development of the Healthcare Professional Council, as well as legislation for every licensure pathway. It is also important to coordinate and collaborate with development partners, especially in countries with limited resources where substantial and technical reliance on external support is common. Therefore, the commitment of the MOH is indispensable.

## Conclusion

The process of ongoing development toward policy evolution for a licensing system in Lao People’s Democratic Republic has been described. This information is expected to enable a broad overview of policy transitions in developing health care professionals, especially those focusing on licensing. In Lao People’s Democratic Republic, the need for licensing systems has increased in recent years due to regional economic integration and a shift in policy toward achieving universal health coverage. A national licensing system would be a significant milestone in health system development, helping to ensure the competency of health care professionals by means of a national examination, continuing professional development, and the revoking of licenses when appropriate. A policy intervention and strengthened governance for continuous development of human resources for health are demanded in order to achieve a health system that can guarantee universal access to health care and social protection to all citizens.
